# Isolated Cysticercosis of Sternocleidomastoid Muscle: Role of Ultrasonography

**DOI:** 10.1155/2021/7102416

**Published:** 2021-09-28

**Authors:** Subash Thapa, Norman Lamichhane, Santosh Joshi

**Affiliations:** ^1^Department of Radiology, Nepal Police Hospital, Kathmandu, Nepal; ^2^Department of Orthopedics, Nepal Police Hospital, Kathmandu, Nepal

## Abstract

Cysticercosis is considered a common healthcare problem, especially in developing countries. The invasion of muscle by the larval stage of the pork tapeworm, *Taenia solium* (i.e., *Cysticercus cellulosae*) usually occurs in association with CNS cysts, concurrent muscle cysts, or both. Isolated skeletal muscle involvement is rare and presents with nonspecific symptoms resulting in a diagnostic dilemma for the treating physician. We report a 20-year-old female with isolated cysticercosis of right sternocleidomastoid muscle presenting as a right neck swelling and mild pain for 4 months, whose diagnosis was established by ultrasonography (USG) and computed tomogram (CT) scan. She was managed conservatively with oral albendazole therapy for four weeks resulting in complete resolution.

## 1. Introduction

Human cysticercosis is an infection caused by the larval stage of the pork tapeworm (*Taenia solium)* [1, 2], which commonly infects the central nervous system, followed by the eyes, subcutaneous tissues, liver, skeletal muscle, lungs, and heart [1, 3]. Muscular cysts are commonly associated with concurrent neurocysticercosis or other muscular cysts or both [4]. Isolated skeletal muscle involvement is rare [2]. Due to the nonspecific symptoms, it presents as a diagnostic dilemma for the treating physician. High-resolution ultrasonography is a noninvasive and real-time diagnostic tool for such a case [2–5].

## 2. Case Report

**Figure 1:**
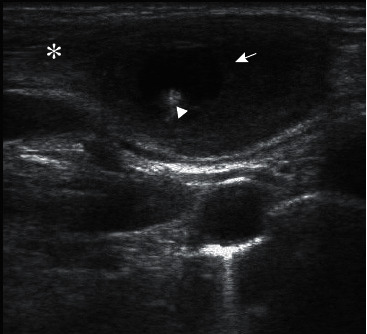
A 20-year-female who presented with gradual neck swelling and mild pain for 4 months. Sagittal grayscale ultrasonography image showing well-defined thick-walled cystic lesion (arrow) in the intramuscular plane in the distal SCM (asterisk) with eccentric hyperechoic foci (arrowhead) within the cavity and surrounding edematous changes.

**Figure 2:**
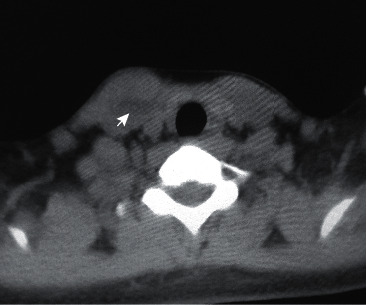
Axial noncontrast computed tomography image of the neck at the level of T1 vertebra showing slightly hypodense cystic lesion with a small area of hyperdensity within the lesion (arrow).

A 20-year-old female presented with complaints of gradually increasing swelling and pain in the right supraclavicular region for four months. Clinical examination showed 2.5 × 1.5 cm firm, mild tender swelling with ill-defined margins in the right supraclavicular area of the neck within the right sternocleidomastoid muscle. The overlying skin did not show any evidence of color change. Examination of the eye, ear, nose, and throat was normal. A complete blood count was within the normal limit. Cysticercosis IgG antibody done by the enzyme-linked immunosorbent assay (ELISA) was negative. Chest X-ray was normal. First day, ultrasonography (USG) showed a well-defined, thick-walled cystic lesion measuring 15 × 7 mm in the intramuscular plane of a right sternocleidomastoid muscle about 2 cm cephalad to the clavicular attachment. An eccentric tiny hyperechoic focus (scolex) was seen within the lesion (Figure 1). Internal vascularity was not present on color or power Doppler interrogation. The right sternocleidomastoid muscle showed decreased perilesional echogenicity, suggestive of edema. Noncontrast computed tomography (CT) scan of the brain was normal; whereas, the neck showed an ill-defined hypodense lesion in the right sternocleidomastoid muscle with a tiny eccentric hyperdense focus within it, which was suggestive of the scolex (Figure 2).

Based on the pathognomonic ultrasonographic and supportive CT scan findings, the diagnosis of cysticercosis of the right sternocleidomastoid muscle was made. The patient was planned for conservative management with an oral albendazole tablet (400 mg twice a day for 28 days) and a tapered dose of steroid. Follow-up USG in 1 month showed relatively ill-defined, small heterogeneous areas in the intramuscular plane of the right sternocleidomastoid muscle, which was significantly small compared to the initial scan. There was complete resolution in the second month.

## 3. Discussion

Cysticercosis among humans was noticed by Parunoli in 1550, though it was first described in pigs by Aristophanes and Aristotle in the 3^rd^ century BC [5]. Human cysticercosis is caused by the infection with the larval stage of the pork tapeworm, *Taenia solium* (i.e., *Cysticercus cellulosae*) [1–4]. Infection by the tapeworm is commonly found in Latin America, sub-Saharan Africa, China, Southern/Southeast Asia, and Eastern Europe regions where the standard of health and sanitation is relatively poor [3, 5].

It is transmitted to humans (definitive host) by ingestion of eggs from contaminated water or food, such as vegetables, or by internal regurgitation of eggs into the stomach released by the adult worm in the intestine. After ingestion of the egg, oncospheres (embryo) in the eggs are released by gastric acid, cross the bowel wall, and enter the bloodstream [2, 3]. The most common location for the lodgement of *Cysticercus* larva is in the brain followed by the eyes, subcutaneous tissue, liver, and skeletal muscles [3]. Muscle involvement usually remains asymptomatic. Most of the cysts remain viable for 10 years and then start degenerating, followed by a vigorous host response [1, 2]. At this stage, the patient complains of symptoms depending on the site of involvement. The natural history of cyst is to resolve by complete resorption or by calcification [3, 6]. Solitary involvement of skeletal muscles is rare; those reported involve the tongue, masseter, lower lip, soft palate, and sternocleidomastoid muscle [7].

The different clinical manifestations involving skeletal muscles are the myalgic, myopathic type, the nodular or mass-like type, and the rare pseudohypertrophy type [3, 6]. The diagnosis of solitary neck swelling is relatively difficult to tease out solely on a clinical basis because the manifestation is nonspecific. Plain X-ray has a less diagnostic role in the early stage but may aid in diagnosis if the cysts are calcified in the late stage (5 years on average) [4, 5, 8], which may have starry sky appearance when cysts are multiple in the subcutaneous or intramuscular plane [9]. Ultrasonography is a readily available noninvasive, nonionizing diagnostic tool in soft tissue imaging [2–10]. The use of a high-frequency linear ultrasound probe (12–15 MHz) is recommended for muscle imaging which can be even performed as an alternative or complement to magnetic resonance imaging (MRI) in many cases [10]. Low-frequency curvilinear probes (2–5 MHz) in contrast are used for greater tissue penetration to determine deep soft tissue pathology at the cost of resolution [10]. High-resolution ultrasonography can demonstrate the presence of eccentric echogenic scolex in the *Cysticercus* within the subcutaneous or muscular plane [2–10] which is pathognomic of cysticercosis and help in establishing the diagnosis with greater confidence [1, 5, 6], as in our case. Four different sonographic appearances of muscular cysticercosis have been described starting from cysticercosis cyst with an inflammatory mass around it, irregular cyst with minimal fluid on the one side, eccentric scolex in the cyst with large irregular collection in adjoining muscle fibers, and calcified cysticercosis [6, 11]; however, confirming the diagnosis by ultrasound is difficult in the absence of salient diagnostic ultrasound features where open or needle biopsy is helpful to determine the etiology before the treatment option [4, 11]. USG also serves as a useful tool in studying the temporal sequence of therapeutic response [1]. CT and MRI scans as noninvasive diagnostic tools can be helpful to define the location, number, and relationship to the surrounding structure [1, 2]. Fine-needle aspiration cytology (FNAC) is considered a confirmatory diagnostic tool of soft tissue cysticercosis [3], as it can identify the tegument layer of the larva [8]; however, the invasive diagnostic method can be avoided with ease after the advancement in the imaging techniques with salient features [3]. Other diagnostic tools including the enzyme-linked immunosorbent assay (ELISA) or enzyme-linked immunoelectrotransfer blot (EITB) as a serological tool can aid in diagnosis. However, immunodiagnostic tests have low sensitivity for the diagnosis of single-lesional cysticercosis, even lower if the lesion is involutive phase or has entered the degenerative process of resolution following antiparasitic treatment or by natural evolution [12]. Also, the presence of antibody in a patient living in the endemic area can misguide the established infection [2]. Soft tissue cysticercosis without abscess can be managed pharmacologically with antihelmenthetics drugs (praziquantel or albendazole) along with steroid [1–6] as in our case; however, cyst with abscess needs surgical excision [2].

## 4. Conclusion

Isolated muscular cysticercosis is a rare clinical entity that should be considered as a differential diagnosis in neck swelling, especially in developing countries. Ultrasonography is a readily available, noninvasive diagnostic tool with pathognomonic findings to establish the diagnosis with greater confidence. Isolated myocysticercosis responds well when treated conservatively by oral antihelmenthetics drugs.

### 4.1. Patient's Perspective

“I was having small tender swelling in my neck for which I visited the doctor. He advised me to do some investigation including an ultrasound before the diagnosis. It was very hard for me to believe that an egg of some live parasites is growing in my body until I saw a round black structure on the ultrasound screen during the scan. It was even terrifying to know that I should undergo some screening to exclude the presence of the egg in other parts of the body including my brain. Hopefully, all the search was negative. I took tablets as prescribed by the doctor, two times a day for about a month, and did my repeat ultrasound scan. Today, I got my third ultrasound scan report without the egg in the report. I would like to thank the doctors' team for all the care and kind words. I am writing to share my experience so that people like me will panic less if they come across what I was going through.”

## Data Availability

The data supporting the results are available on request to the authors.
